# Mucosal expression of Ca and P transporters and claudins in the small intestine of broilers is altered by dietary Ca:P in a limestone particle size dependent manner

**DOI:** 10.1371/journal.pone.0273852

**Published:** 2022-09-01

**Authors:** Y. X. Hu, J. van Baal, W. H. Hendriks, M. Duijster, M. M. van Krimpen, P. Bikker

**Affiliations:** 1 Wageningen Livestock Research, Wageningen University & Research, Wageningen, the Netherlands; 2 Wageningen University & Research, Animal Nutrition Group, Wageningen, the Netherlands; 3 R&D Department, De Heus Animal Nutrition B.V, Ede, the Netherlands; Tokat Gaziosmanpasa Universitesi, TURKEY

## Abstract

High calcium (Ca) intake and fine limestone reduces precaecal phosphorus (P) absorption independently of P solubility in broilers. This study aimed to determine whether dietary total Ca: total P ratio (Ca:P) and limestone particle size (LPS) affect gene expression of P transporters in the small intestine. A total of 384 one-day-old Ross 308 male broiler chickens received diets low (0.50), medium (1.00) or high (1.75) in Ca:P containing either fine (160 μm) or coarse (1062 μm) limestone, in a 3×2 factorial arrangement. Expression of Ca- and P-related genes were determined using real-time quantitative PCR (RT-qPCR) in duodenum and jejunum. Increasing dietary Ca:P decreased duodenal calcium-sensing receptor (CaSR), calbindin-D28k (CaBP-D28k), plasma membrane Ca-ATPase 1 (PMCA1) and sodium-coupled P cotransporter type IIb (NaPi-IIb), but not transient receptor potential canonical 1 (TRPC1) mRNA. This effect was greater with fine limestone when Ca:P increased from low to medium, but greater with coarse limestone when increased from medium to high. A similar inhibitory effect was observed for jejunal CaBP-D28k expression where increasing dietary Ca:P and fine limestone decreased CaSR mRNA, while dietary Ca:P decreased TRPC1 mRNA only for coarse limestone. It also decreased jejunal NaPi-IIb mRNA irrespective of LPS. Dietary treatments did not affect jejunal PMCA1 mRNA expression or that of inorganic phosphate transporter 1 and 2 and xenotropic and polytropic retrovirus receptor 1 in both intestinal segments. Dietary Ca increase reduced mucosal claudin-2 mRNA in both segments, and jejunal zonula occludens-1 (ZO-1) mRNA only for coarse limestone. In conclusion, increasing dietary Ca:P reduced expression of duodenal P transporters (NaPi-IIb) in a LPS dependent manner, hence Ca induced reduction in intestinal P absorption is mediated by decreasing P transporters expression. Dietary Ca reduces Ca digestibility by downregulating mRNA expression of both Ca permeable claudin-2 and Ca transporters (CaBP-D28k, PMCA1).

## Introduction

Calcium (Ca) and phosphorus (P) are two macro minerals involved in multiple biological processes, including cell signalling, synaptic transmission, muscle contraction, bone mineralization and many other biochemical reactions in both humans and animals [1]. According to a survey based on 795 broiler and pig diets from 2010–2015 [2], the analysed Ca content is on average 2.2 g/kg higher than the formulated Ca content, probably due to a lack of knowledge on variation in Ca content of feed ingredients. It is generally accepted that free Ca ions reduce intestinal P absorption by the formation of insoluble Ca-P and Ca-phytate complexes [3], although the presence of such complexes has still not been demonstrated in digesta of animals. Experimental data showed that reduction of dietary total Ca: total P ratio (Ca:P) improves intestinal P absorption but may in the case of excessive reduction compromise growth performance of broilers. In our recent study [4], aimed to clarify the impact of dietary Ca:P combined with limestone particle size on intestinal Ca and P digestibility in broilers, increasing dietary Ca:P linearly reduced apparent P absorption in the bird’s gastrointestinal tract (GIT). Strikingly, P solubility was not affected by dietary Ca level or limestone particle size in digesta collected from the crop, jejunum or ileum. These findings indicate that luminal Ca-P or Ca-phytate complexation are not the only mechanisms for the reduction in intestinal P absorption in response to high dietary Ca:P. Uptake of Ca and P by enterocytes occurs through transcellular and paracellular pathways, which are orchestrated predominantly by the parathyroid hormone (PTH), fibroblast growth factor 23 (FGF-23) and vitamin-D_3_ [5, 6]. Together with our previous observation that fine limestone is better digestible than coarse limestone [4], it was hypothesised that intake of high dietary Ca:P in broilers reduces intestinal P absorption by reducing the local gene expression of P transporters and that this inhibitory effect is greater for fine compared to coarse limestone.

It is presumed that the molecular mechanisms for Ca and P absorption in chickens are rather similar to those of mammals due to the high homology of candidate genes involved [7]. The transcellular route is an overall active, saturated process, which requires the entry of ions through protein carriers in the apical plasma membrane, intracellular diffusion and extrusion via transporters in the basolateral membrane [8]. Regarding intestinal absorption and renal reabsorption of P, apical P uptake is considered to be mediated by members of the sodium (Na)-coupled P cotransporter family, predominantly Na-coupled P transporter type IIb (NaPi-IIb/SLC34A2) which was shown to contribute to over 90% of total active P absorption in the digestive tract of mice [9]. Contribution of NaPi-IIb to intestinal P absorption in chick jejunum was confirmed by Huber et al. [10], who observed that uptake of P into jejunal brush boarder vesicles was highly dependent on Na^+^ but not potassium (K^+^). A possible role of inorganic phosphate transporter 1 (PiT-1/SLC20A1) and/or inorganic phosphate transporter 2 (PiT-2/SLC20A2) has also been postulated, but its relevance for whole body P homeostasis remains elusive [11, 12]. In respect to basolateral P extrusion, some evidence put forward xenotropic and polytropic retrovirus receptor 1 (XPR1) as a novel candidate for basolateral P exportation in animals [13, 14]. As for Ca, the ion first enters the intestinal epithelial cell across the apical membranes passively through transient receptor potential cation channel subfamily V 5 or 6 (TRPV5 or TRPV6) [15]. This is followed by binding of Ca to calbindin proteins (in birds exclusively calbindin D28k (CaBP-D28k)) and transferred to the basolateral membrane. Involvement of CaBP-D28k in intestinal Ca absorption in chicks intestine has been confirmed, since a good correlation was obtained between the concentration of CaBP-D28k and the rate of Ca absorption [16]. Basolateral exit is then driven by the plasma membrane Ca-ATPase 1 (PMCA1) and/or Na^+^/Ca^2+^ exchanger (NCX1) [17] against the electrochemical gradient and thus by active transport. Deletion of intestinal PMCA1 in mice [18] was shown to be associated with a decreased bone mineral density and impaired responsiveness to 1,25-dihydroxycalciferol (1,25(OH)_2_D_3_). Recently, transient receptor potential canonical 1 (TRPC1) is put forward as a novel candidate apical Ca transporter as its expression level increases upon maturity in layers [19].

Compared to transcellular transport, the mechanisms involved in the paracellular routes for Ca and P are far less understood. Increasing evidence indicates that certain members of the claudin (CLDN) family control the paracellular permeability of the tight junctions in epithelial cells by forming a selective seal (i.e. barrier) or pore (i.e. gate) for ions in tight junctions (TJ) [20]. Indeed, in mice CLDN-2, -12, and -15 are associated with intestinal Ca uptake [21], while CLDN-1 and -5 are linked to sealing functions that could diminish Ca transport [22, 23]. Unfortunately, the literature is devoid of information demonstrating the importance on the expression pattern of TJ proteins in the broiler’s GIT in response to dietary intake levels of Ca and/or P.

The objective of the present study was to investigate the influence of increasing dietary Ca:P and limestone particle size on the mRNA expression levels of transporters and TJ proteins related to Ca and P absorption in two segments of the small intestinal tract, i.e. duodenum and jejunum of broilers in order to obtain a better understanding of the consequences of such diets on the broiler’s Ca and P homeostasis.

## Experimental methods

The experiment was conducted in the broiler research accommodation of De Heus (Eerde, the Netherlands). All procedures complied with the Dutch law on animal experiments in accordance with EU directive 2010/63. The study was approved by the animal research Ethical Committee of Wageningen University & Research, Wageningen, the Netherlands (no. 2016.D-0065.004).

### Animals, experimental design and tissue collection

This research is a successive study to that of Hu et al. [4] concerning the interactive *in vivo* effects of limestone particle size and dietary Ca:P on intestinal Ca and P digestibility coefficient, growth performance and tibia breaking strength in broilers. Details regarding the experimental design, feed composition, animal husbandry, feeding regime, and sample collection can be found in our previous article [4]. Briefly, 384 one-day-old Ross 308 broiler chickens had *ad libitum* access to a standard commercial starter feed from day 0–13. From day 14 onwards, birds received one of six diets in a 3×2 factorial arrangement containing one of three Ca:P (0.50, 1.00, and 1.75) feeds either with fine or coarse limestone (geometric mean diameter 160 or 1062 μm). The intended Ca content was 2.7, 5.5 and 9.6 g/kg for the low, medium and high Ca:P, respectively, which was below, at and above the minimal Ca requirement for chickens, respectively. The intended P content was fixed at 5.5 g/kg for all Ca:P diets. Each treatment was replicated four times with 16 birds per replicate pen. Fine and coarse limestone were separated from the same batch of product (Sibelco, Maastricht, the Netherlands) via sieving through a 500 μm screen and supplemented to a low-calcium phytase-free basal diet with corn, wheat and soybean meal as major ingredients. Monocalcium phosphate and monosodium phosphate were the main sources of P. The broilers had free access to the pelleted diets and drinking water.

On day 20 and 21, three birds per pen were randomly selected from 12 out of the 24 pens, sedated by a mixture of sedadum and ketamine and then killed by electronic stunning and exsanguination. After cleansed in water, the mucosa was scraped from the middle of the duodenum and jejunum, immediately frozen in liquid nitrogen and stored at -80 °C until further analysis. These two gut segments were used for analysis of mRNA abundance of genes related to trans- and paracellular Ca and P absorption since on average, over 85% of the precaecal digestible Ca and P was absorbed proximal of the ileum [4]. The remaining birds were kept to day 39 at which time they reach the commonly used commercial slaughter weight, shipped to a nearby slaughter house and slaughtered according to standard procedures. Health and wellbeing of the animals was monitored and registered daily. Particular attention was given to the gait of the birds. Furthermore, the availability of feed and water was checked twice a day.

### Real-time quantitative PCR

Gene expression was analysed on individual bird level. Deep-frozen intestinal mucosa samples were ground in liquid nitrogen and total RNA was isolated with TRIzol (ThermoFisher Scientific, Waltham, MA) according to the manufacture’s instruction. Isolated RNA was subjected to on-column DNase treatment to remove possible genomic DNA contamination with the Nucleospin II kit (Macherey Nagel). Quantity and integrity of RNA were determined with the NanoDrop 1000 Spectrophotometer (ThermoFisher Scientific) and 2100 Bioanalyzer and RNA 6000 Nano LabChip kit (Agilent Technologies), respectively. Five hundred ng RNA was reverse transcribed with a Superscript III kit (ThermoFisher Scientific) and mRNA levels were assessed by real-time quantitative PCR (RT-qPCR) amplification on a QuantStudio 5 Real-Time PCR System (ThermoFisher Scientific) using the SensiFAST^™^ SYBR^®^ low-ROX Kit (Bioline) under the conditions: 95 °C for 15 s and 60 °C for 30 s for 40 cycles. This was followed by a melting curve analysis ramping from 60 to 95 °C with a rate of 0.1 °C/s in order to compare the melting temperature between standards and samples, thereby confirming PCR specificity. The used primer sequences were designed with Primer Express Software (Life Technologies, Bleiswijk, the Netherlands), and where possible recommended primer sets that span an intron were selected and presented in Table 1. Absolute quantitative mRNA measurement was performed by establishing a linear calibration curve using 10-fold serial dilutions of cDNA template for corresponding genes. All primers had a minimal amplification efficiency of 89%. Using the standard curve, the CT value of target genes was converted to absolute scale. Since the Normfinder [24] algorithm demonstrated that the combination of two reference genes, importin 8 (IPO8) and eukaryotic translation elongation factor 2 (EEF2), was most stable among other candidates (beta-actin (ACTB), glyceraldehyde 3-phosphate dehydrogenase (GAPDH), 60S acidic ribosomal protein P0 (RPLP0)), expression levels were normalized to the geometric mean of IPO8 and EEF2.

**Table 1:** Primers used for real-time quantitative PCR (RT-qPCR) analysis.

Gene	Sense 5’-3’	Antisense 5’-3’	Accession no.
**Ca transporters**			
**CaSR**	GCCAATCTGCTGGGACTCTT	CTGATGCTCGTCATTGGGGA	XM_416491.6
**CaBP-D28k**	CGAGATCTGGCACCACTACG	ACCTGAGCAAGCTCAACGAT	NM_205513.1
**PMCA1**	ACTCTGATGGCAGTTTCCGA	GTCACGGTCCCTAGGTCTGA	NM_001168002.3
**TRPC1**	GAATACGATGGGACCAGCCC	AGCTGATTGCGTGACTCTTCT	NM_001004409.2
**TRPV6**	GACCAGAGCAAAGAGGGACC	CCGCCTCTGCATGAGGTATT	XM_004938143.3
**P transporters**			
**NaPi-IIa**	GAAGCCAGGTGCCTCTGATG	AGAGGATGGCGTTGTCCTTG	XM_015293846.2
**NaPi-IIb**	TGGCTTTGTCCCTGCTTGTT	CCAGCCAGCCAAGTAAAAGG	NM_204474.2
**PiT-1**	TGAAGCTTCCCATCTCGGGT	AGGACAACACGATTTTTAGCAGC	XM_015297502.2
**PiT-2**	GCTGGGAGCAAAAGTAGGAGA	AAACAGCAGAACCAACCATCG	NM_001305398.1
**XPR1**	AACCTGGAGACAACACGAGG	CGTTGGTCACCACTTCCTCT	XM_422258.6
**Tight junction proteins and VDR**			
**CLDN-2**	CAACTGGAAGATCAGCTCCT	TGTAGATGTCGCACTGAGTG	NM_001277622.1
**CLDN-12**	CTCTTATTCCTCCTCGCATG	GTCAAAGCTAAAGACAGGCT	XM_025148431.1
**CLDN-16**	GGGATCCAAACATGTGATGA	AGAGAAATCCAAATCCTGCC	XM_426702.4
**ZO-1**	CCGCAGTCGTTCACGATCT	GGAGAATGTCTGGAATGGTCTGA	XM_015278981.2
**VDR**	GGCTCAGGTTTTGCAGATTTG	CAGCATCGCCTTTCCCATT	NM_205098.1
**Reference genes**			
**ACTB**	GCCCTGGCACCTAGCACAAT	GCGGTGGACAATGGAGGGT	NM_205518.1
**EEF2**	CAGTTGGCTTTGGTTCTGGC	AAAGTATCTGTCTCCCCACAGC	NM_205368.1
**GAPDH**	ATCCCTGAGCTGAATGGGAAG	AGCAGCCTTCACTACCCTCT	NM_204305.1
**IPO8**	ACCTCCGAGCTAGATCCTGT	GGCTCTTCTTCGCCAACTCT	XM_015287054.2
**RPLP0**	TTGGGCATCACCACAAAGATT	CCCACTTTGTCTCCGGTCTTAA	NM_204987.2

ACTB, beta actin; CaBP-D28k, calbindin D28k; CaSR, calcium sensing receptor; CLDN, claudin; EEF2, eukaryotic translation elongation factor 2; GAPDH, glyceraldehyde 3-phosphate dehydrogenase; IPO8, importin 8; NaPi-IIa, sodium dependent phosphate transporter IIa; NaPi-IIb, sodium dependent phosphate transporter IIb; PiT-1, inorganic phosphate transporter 1; PiT-2, inorganic phosphate transporter 2; PMCA1, plasma membrane calcium-ATPase 1; RPLP0, 60S acidic ribosomal protein P0; TRPC1, transient receptor potential canonical 1; TRPV6, transient receptor potential cation channel subfamily V member 6; VDR, vitamin-D_3_ receptor; XPR1, xenotropic and polytropic retrovirus receptor 1; ZO-1, zonula occludens-1. The expression levels of gene of interest were normalized to the geometric mean of IPO8 and EEF2 (see experimental methods).

### Statistical analysis

Pens in which three birds were selected for gene expression analysis were used as experimental unit for statistical analysis. Data were subjected to a two-way ANOVA using the MIXED procedure of SAS 9.4 (SAS Institute, Cary, NC). The model included limestone particle size and Ca:P (main effects) and their interaction as fixed effect. With this procedure the mean value of three animals in each pen was used for the statistical analysis. Distribution and variance homogeneity of Studentized residuals were visually checked via graphics plotted using ODS GRAPHICS function. The LSMEANS procedure with a PDIFF option was used to estimate the difference between means. Probability was considered significant at *P*≤0.05 and a trend at 0.05<*P*≤0.1.

## Results

The realized Ca and P content in diets was close to the intended values, with an analysed Ca:P of 0.54, 1.08 and 1.78 for the fine limestone, and 0.53, 0.99 and 1.73 for the coarse limestone, respectively. During the whole experimental period, the birds grew well and realized a high feed intake and growth rate. Average body weight at the end of the experiment met or exceeded the performance objectives of the breeding company [25].

### Duodenum

The RT-qPCR analysis revealed that the transcript level of the extracellular calcium-sensing receptor (CaSR) was reduced in the duodenal mucosa of broilers with a dietary Ca:P increase from low (0.5) to high (1.75) in combination with fine limestone, but was reduced only when dietary Ca:P was increased from low to medium (1.00) in the presence of coarse limestone (*P*_interaction_ = 0.047, Table 2 and S1 Fig in S1 File). In addition, increasing dietary Ca:P also downregulated mRNA expression of Ca-ATPase PMCA1 and CaBP-D28k (*P*<0.001) in this segment. However, the latter reduction was found with fine limestone when dietary Ca:P was increased from low to medium, but with coarse limestone from medium to high (*P*_interaction_ = 0.016 and 0.037, respectively). Moreover, expression of TRPC1 was not affected by either dietary Ca or limestone particle size. Similarly, an interaction effect between Ca:P and limestone particle size was observed for the Na-P cotransporter NaPi-IIb (*P*_interaction_ = 0.035), with a reduction in its expression level when dietary Ca:P was increased from low to medium together with fine limestone but with coarse limestone only from medium to high. Expression of the other Na-P cotransporters PiT-1 and PiT-2, and the putative basolateral P channel XPR1 were not affected by the dietary treatments. As for TJ proteins, CLDN-2 was downregulated with high dietary Ca intake (*P*<0.001) irrespective of limestone size (no interaction effect). Expression of zonula occludens-1 (ZO-1) and CLDN-12, however, remained unaffected by dietary treatments. The same was true for the transcript level of vitamin D_3_ receptor (VDR). We did not detect expression of NaPi-IIa, TRPV6 or CLDN-16 in the broiler’s duodenal mucosa.

**Table 2:** Least square mean of mRNA expression levels of Ca and P transporters and claudins in the duodenal mucosa in broilers as affected by dietary total Ca: Total P ratio (Ca:P), limestone particle sizes and their interaction ^1^^,^^2^^,^^3^.

Limestone	Ca:P	Ca transporters				P transporters				Tight junctions and VDR			
		CaSR	TRPC1	CaBP-D28k	PMCA1	NaPi-IIb	PiT-1	PiT-2	XPR1	ZO-1	CLDN-2	CLDN-12	VDR
**Fine**	**0.50**	0.18^a^	1.29	1319^a^	478^a^	2652^a^	13.0	1.47	12.8	1.89	108	1.77	2.59
	**1.00**	0.12^b^	1.30	921^cd^	373^b^	2054^bc^	11.3	1.46	13.1	1.98	93	1.88	2.54
	**1.75**	0.07^c^	1.27	998^bcd^	371^b^	2173^abc^	11.3	1.20	13.2	1.93	77	1.88	3.04
**Coarse**	**0.50**	0.23^a^	1.29	1248^ab^	410^b^	2519^ab^	11.8	1.24	12.8	2.01	107	1.83	2.64
	**1.00**	0.11^bc^	1.24	1144^abc^	412^b^	2633^a^	12.6	1.43	12.7	1.88	93	1.79	3.00
	**1.75**	0.11^bc^	1.26	754^d^	311^c^	1875^c^	12.9	1.44	12.9	1.84	76	1.85	2.76
**SEM**		0.020	0.084	125.4	27.7	245.9	1.73	0.252	0.86	0.094	6.0	0.095	0.263
**Ca:P mean**													
**0.50**		0.21	1.29	1283	444	2586	12.4	1.36	12.8	1.95	108^a^	1.80	2.62
**1.00**		0.12	1.27	1032	393	2344	12.0	1.44	12.9	1.93	93^b^	1.84	2.77
**1.75**		0.09	1.27	876	341	2024	12.1	1.32	13.1	1.88	76^c^	1.86	2.90
**SEM**		0.014	0.059	88.7	19.6	173.9	1.22	0.141	0.61	0.066	4.2	0.067	0.186
**Limestone mean**													
**Fine**		0.13	1.27	1079	408	2293	11.9	1.38	13.0	1.93	93	1.84	2.72
**Coarse**		0.15	1.26	1049	378	2343	12.5	1.37	12.8	1.91	92	1.82	2.80
**SEM**		0.012	0.049	72.4	16.0	142.0	1.00	0.115	0.50	0.054	3.5	0.055	0.152
***P*-value**													
**Ca:P**		<0.001	0.913	<0.001	<0.001	0.009	0.926	0.671	0.907	0.593	<0.001	0.651	0.335
**Limestone**		0.030	0.629	0.673	0.068	0.727	0.561	0.962	0.609	0.707	0.853	0.652	0.651
**Ca:P×limestone**		0.047	0.914	0.037	0.016	0.035	0.463	0.265	0.926	0.170	0.976	0.417	0.151

^1^ Determined using absolute quantification and normalized by the geometric mean of eukaryotic translation elongation factor 2 and importin 8.

^2^ Means of 4 pens (experimental unit) per treatment and 3 birds per pen.

^3^ Fine and coarse limestone were separated from the same product via sieving (Sibelco, the Netherlands; geometric mean diameter 160 vs. 1062 μm).

^a-d^ Values lacking a common superscript within a column differ (*P*≤0.05).

CaBP-D28k, calbindin D28k; CaSR, calcium sensing receptor; CLDN, claudin; NaPi-IIa, sodium dependent phosphate transporter IIa; NaPi-IIb, sodium-coupled phosphate transporter type IIb; PiT-1, inorganic phosphate transporter 1; PiT-2, inorganic phosphate transporter 2; PMCA1, plasma membrane calcium-ATPase 1; RPLP0, 60S acidic ribosomal protein P0; TRPC1, transient receptor potential canonical 1; TRPV6, transient receptor potential cation channel subfamily V member 6; VDR, vitamin-D_3_ receptor; XPR1, xenotropic and polytropic retrovirus receptor 1; ZO-1, zonula occludens-1. Of note, NaPi-IIa, TRPV6 and CLDN-16 mRNA were not detectable.

### Jejunum

Jejunal mucosal expression of CaSR was reduced with high dietary Ca:P (*P*<0.001) and with fine limestone intake (*P* = 0.002, Table 3). An interaction effect was observed for CaBP-D28k mRNA levels due to the fact that the inhibitory effect was greatest with fine limestone at an increase of Ca:P from low to medium but with coarse limestone from medium to high (*P*_interaction_ = 0.004, S2 Fig in S1 File). Contrary to findings in the duodenum, PMCA1 mRNA levels remained unaffected by the dietary treatments in the jejunum. Besides, dietary Ca increment reduced jejunal expression of TRPC1 but only for diets supplied with coarse limestone (*P*_interaction_ = 0.05). Increasing dietary Ca:P also reduced CLDN-2 gene expression levels (*P*<0.015) independent of limestone size. Compared to the low Ca:P group, high dietary Ca:P downregulated zonula occludens-1 (ZO-1) mRNA levels only in the presence of coarse limestone (*P*_interaction_ = 0.047). The CLDN-12 mRNA tended to be higher in broilers fed the medium Ca:P diet combined with fine limestone (*P*_interaction_ = 0.066). The VDR mRNA levels were not influenced by the dietary treatments. Regarding P absorption, NaPi-IIb expression was downregulated by increasing dietary Ca:P (*P* = 0.004) regardless of the addition of the different limestone sizes. Similarly to duodenal mucosa, jejunal mucosal transcript levels of PiT-1, PiT-2 and XPR1 were not affected in response to the dietary treatments (*P*>0.05; Table 3) and the levels of NaPi-IIa, TRPV6 and CLDN-16 were below the limit of detection.

**Table 3:** Least square mean of mRNA expression levels of Ca and P transporters and claudins in the jejunal mucosa as affected by dietary total Ca: Total P ratio (Ca:P), limestone particle sizes and their interaction in broilers ^1^^,^^2^^,^^3^.

Limestone	Ca:P	Ca transporters				transporters				Tight junctions and VDR			
		CaSR	TRPC1	CaBP-D28k	PMCA1	NaPi-IIb	PiT-1	PiT-2	XPR1	ZO-1	CLDN-2	CLDN-12	VDR
**Fine**	0.50	0.94	1.47^b^	668^a^	356	1276	16.0	1.26	8.31	2.42^abc^	70.1	1.85	1.71
	1.00	0.70	1.61^ab^	418^c^	287	971	15.1	1.01	8.01	2.54^ab^	51.2	2.03	1.71
	1.75	0.43	1.41^b^	530^b^	330	952	16.0	0.92	8.56	2.35^b^	45.5	1.85	2.10
**Coarse**	0.50	1.14	1.69^a^	664^a^	327	1282	16.2	1.10	8.40	2.64^a^	65.4	2.02	2.17
	1.00	0.95	1.44^b^	561^ab^	320	1296	16.0	1.15	8.58	2.41^bc^	56.1	1.81	1.94
	1.75	0.65	1.43^b^	401^c^	295	846	14.7	1.12	8.37	2.27^c^	45.6	1.83	2.00
**SEM**		0.115	0.109	53.8	36.1	151.8	2.81	0.137	0.617	0.100	6.28	0.111	0.229
**Ca:P mean**													
**0.50**		1.04^a^	1.58	666	342	1279^a^	16.1	1.18	8.35	2.53	67.8^a^	1.94	1.94
**1.00**		0.83^b^	1.53	489	304	1133^a^	15.5	1.08	8.30	2.47	53.6^b^	1.92	1.82
**1.75**		0.54^c^	1.42	466	312	899^b^	15.3	1.02	8.46	2.31	45.6^b^	1.84	2.05
**SEM**		0.081	0.077	38.1	25.5	107.4	1.99	0.097	0.436	0.071	4.44	0.078	0.163
**Limestone mean**													
**Fine**		0.69	1.50	539	324	1066	15.7	1.06	8.30	2.43	55.7	1.91	1.84
**Coarse**		0.92	1.52	542	314	1141	15.6	1.12	8.45	2.44	55.6	1.89	2.04
**SEM**		0.066	0.063	31.1	20.8	87.6	1.63	0.079	0.356	0.058	3.63	0.064	0.133
***P*-value**													
**Ca:P**		<0.001	0.125	<0.001	0.308	0.004	0.925	0.222	0.927	0.015	<0.001	0.451	0.420
**Limestone**		0.002	0.738	0.915	0.626	0.398	0.972	0.510	0.704	0.891	0.973	0.604	0.135
**Ca:P×limestone**		0.949	0.050	0.004	0.351	0.125	0.867	0.153	0.677	0.047	0.553	0.066	0.271

^1^ Determined using absolute quantification normalized by eukaryotic translation elongation factor 2 and importin 8.

^2^ Means of 4 pens (experimental unit) per treatment and 3 birds per pen.

^3^ Fine and coarse limestone were separated from the same product via sieving (Sibelco, the Netherlands; geometric mean diameter 160 vs. 1062 μm).

^a-c^ Values lacking a common superscript within a column differ (*P*≤0.05).

CaBP-D28k, calbindin D28k; CaSR, calcium sensing receptor; CLDN, claudin; NaPi-IIa, sodium dependent phosphate transporter IIa; NaPi-IIb, sodium-coupled phosphate transporter IIb; PiT-1, inorganic phosphate transporter 1; PiT-2, inorganic phosphate transporter 2; PMCA1, plasma membrane calcium-ATPase 1; RPLP0, 60S acidic ribosomal protein P0; TRPC1, transient receptor potential canonical 1; TRPV6, transient receptor potential cation channel subfamily V member 6; VDR, vitamin-D_3_ receptor; XPR1, xenotropic and polytropic retrovirus receptor 1; ZO-1, zonula occludens-1. Of note, TRPV6, NaPi-IIa and CLDN-16 mRNA were not detectable.

## Discussion

This study aimed to investigate the influence of dietary Ca:P and limestone particle size on the mRNA expression of transporters and TJ proteins related to Ca and P absorption in the duodenum and jejunum of broilers. The data in the present study demonstrates that high dietary Ca:P reduces duodenal mRNA level of NaPi-IIb in broilers to a greater extent in the presence of fine limestone than coarse limestone. The inhibitory effect on the jejunal NaPi-IIb mRNA level, however, appears to be independent of limestone particle size. Similarly, in the duodenum, increasing dietary Ca:P downregulated the expression of the Ca absorption-related genes CaBP-D28k and PMCA1 in a limestone size dependent manner, but this dependency was not seen for the latter gene in the jejunum. Irrespective of limestone particle size, the expression of CLDN-2 in both intestinal segments was linearly downregulated with increasing dietary Ca:P.

An intriguing observation is that our RT-qPCR procedure did not detect any substantial gene expression of TRPV6 in the duodenal and jejunal mucosa, while ample studies have demonstrated that TRPV6 is responsible for the highly selective apical uptake of Ca by absorptive epithelial cells in mammals. The expression of TRPV6 in the chicken’s intestine is controversial; the absence of TRPV6 mRNA in the chicken small intestine is also reported by others [7, 26, 27], while the presence is supported by the evidence of an immunoreactive protein in the small intestine of chickens [26, 28]. Gloux et al. [19] postulated that TRPC1 may be a new candidate Ca transporter which also allows apical Ca entry in the gut of chickens. Indeed, we observed a downregulation of TRPC1 mRNA expression in response to an increase in dietary Ca combined with coarse limestone in jejunal but not duodenal mucosa. However, expression of TRPC1 was not regulatable when the diet contained fine limestone. Further studies are thus required to establish the exact transport system for Ca by the intestinal epithelium of chickens.

Our observation that high dietary Ca:P downregulates expression of NaPi-IIb, CaBP-D28k and PMCA1 in the duodenum and jejunum in broilers is in agreement with studies in mammals. We previously reported that in these broilers, increasing dietary Ca:P linearly increased serum Ca concentration, accompanied by a numerical decrease in serum 1,25(OH)_2_D_3_ and apparent ileal Ca digestibility [4]. The VDR was expressed in the small intestine and unaffected by the dietary treatments. The observed lowering of CaBP-D28k, PMCA1 and NaPi-IIb mRNA levels may be ascribed to the decrease in 1,25(OH)_2_D_3_ concentration since these genes contain a responsive element on their promoter sequence [7].

Since NaPi-IIb is shown to be responsible for over 90% of the transcellular P absorption in the digestive tract of mice [9], and P entry into epithelial cells via NaPi-IIb is considered as the rate-limiting step in the process of P absorption across the epithelial cells [29], its downregulation with high Ca:P indicates a lower capacity of P absorption by both duodenal and jejunal segment. This notion is in line with our previous observation [4] that high dietary Ca:P reduced serum P levels in these broilers. Moreover, we previously reported that increasing dietary Ca:P from 0.50 to 1.75 decreased apparent distal ileal Ca digestibility by approximately 30% [4], which supported our result that jejunal NaPi-IIb mRNA was 30% higher in broilers fed the low compared to high Ca:P diet. In another recent broiler study from our group (data not published), an increase in dietary Ca also reduced serum P accompanied with a downregulation of duodenal expression of NaPi-IIb, PiT-2 and XPR1, which is in line with results obtained in the present study. Furthermore, several studies [11, 29] have shown that expression of NaPi-IIb was regulated at posttranscription level, i.e. trafficking of NaPi-IIb to and from the apical membrane via its PDZ binding motif. However, we did not further investigate the posttranscription regulation of NaPi-IIb since it was beyond the scope of this study.

We also observed that the limestone particle size modulated the effect of dietary Ca:P on the duodenal expression of NaPi-IIb, CaBP-D28k and PMCA1. Compared to the low Ca:P diet, mRNA levels of these genes were lower only in broilers fed a high Ca:P diet when coarse limestone was used, while they were lower in both high and medium Ca:P for the fine limestone diets. An explanation is that fine limestone has a higher rate of solubilisation [30], providing a greater Ca availability for absorption in the duodenum [4]. Combined with a medium Ca:P level, this may lead to an earlier overload of the Ca transcellular pathway compared to the combination with coarse limestone. The epithelial cells will prevent excessive long term cellular uptake by reducing expression of these three genes to lower the active Ca and P transport capacity in the duodenum. Because of the lower rate of solubilisation of coarse limestone, the slower release of Ca^2+^, and the gradual Ca^2+^ absorption overtime, coarse limestone was less likely to cause a systemic Ca^2+^ overload in the animals. Noteworthy is that we previously demonstrated that serum Ca, P and 1,25(OH)_2_D_3_ were independent of limestone particle size [4], which is probably because the broilers were under steady-state conditions (realized via providing constant lighting in the four days before and during sample collection) thus representing intestinal Ca and P absorption from the total tract rather than the proximal part of the gut. Furthermore, an acute release of Ca from fine limestone could activate extracellular CaSR, thereby, directly downregulating expression of Ca transporters independent of serum circulation of PTH or 1,25(OH)_2_D_3_ [31].

Increasing dietary Ca:P might also reduce paracellular Ca permeation. No information is available about the intestinal Ca absorption via the paracellular route in broilers. In mammals, Ca permeation was considered to be stable and independent of 1,25(OH)_2_D_3_ [32, 33]. However, in Caco-2 cells, it has been demonstrated that CLDN-2 formed pores selective for Ca ions, thus increasing Ca permeation across the monolayer and its expression level was upregulated after 1,25(OH)_2_D_3_ stimulation [34]. In the present study, we found a significant reduction of mRNA expression of CLDN-2 in the broilers evoked by high dietary Ca:P intake in both duodenum and jejunum, which could also be regulated by 1,25(OH)_2_D_3_. This is in agreement with the numerical drop of serum 1,25(OH)_2_D_3_ levels in these broilers [4] and the presence of a responsive element to vitamin D_3_ in the promoter of the CLDN-2 gene [35]. In support of this notion is the reduced CLDN-2 expression by a high dietary Ca:P in these gut segments (Tables 2 and 3) while the serum 1,25(OH)_2_D_3_ levels were not affected by limestone particle size [4]. The lower mRNA expression of CLDN-2 probably attributes to a decreased capacity of intestinal paracellular Ca permeation. Of note is that this reduction in CLDN-2 expression might also impact intestinal leakiness in broilers fed a high Ca diet. Unfortunately, CLDN that may facilitate paracellular P permeation in animals have not been identified yet and awaits, therefore, further studies.

Our previous study demonstrated that over 85% of precaecal digestible Ca and P is absorbed proximal to the ileum [4]. Here, we report that the duodenal mucosa displayed a higher expression level of NaPi-IIb, CaBP-D28k and PMCA1 than the jejunal mucosa, which is in line with our previous finding [4]. Considering that active apical entry of P [29] and intracellular diffusion of Ca [36] are regarded as the rate-limiting processes for active translocation of Ca and P across the intestinal epithelial cell, a gradual reduction of NaPi-IIb and CaBP-D28k may indicate a lower contribution of active Ca and P absorption along the small intestine. Indeed, by using *in situ* ligated intestinal loops infused with solutions varying in P concentration, Liu et al. [37] confirmed that P absorption is a carrier-mediated process in the duodenum and a passive unsaturated process in the jejunum and ileum. It should be noted that an abundant expression of Ca and P transporters does not necessarily mean substantial absorption of Ca and P in the duodenum, as our previous broiler study [4] showed that digesta mean retention time was rather short in this segment (approximately 1 min) hence limiting the contribution of this segment to intestinal Ca and P absorption.

Whole body Ca and P homeostasis is maintained via delicate regulation of Ca and P (re)absorption in both GIT and kidney. Expression of the Ca and P transporters and claudins in the kidney of these broilers, however, was not measured in the present study. It is conceivable that a high dietary Ca intake reduced intestinal P absorption at least partly via reducing mRNA expression of P transporters in the duodenal and jejunal mucosa, while it probably would enhance expression of P transporters in the kidney in order to reduce urinary P excretion and maintain whole body Ca and P balance. This hypothesis, however, need more experimental evidence to confirm.

In conclusion, increasing dietary Ca intake reduces expression of P transporters (NaPi-IIb) in a limestone particle size dependent manner in the duodenal mucosa. This supports our hypothesis that the Ca induced reduction in intestinal P absorption in broilers is mediated by an effect on mRNA expression of P transporters. Expression of Ca permeable claudin (CLDN-2) and Ca transporters (CaBP-D28k, PMCA1) is also downregulated with dietary Ca increase, suggesting that the reduction in Ca digestibility with increasing Ca intake is mediated by the effect of dietary Ca on these Ca transporting proteins.

## Supporting information

S1 File Supplementary figures. (PDF)
