# Supplementary material for: Mucosal expression of Ca and P transporters and claudins in the small intestine of broilers is altered by dietary Ca:P in a limestone particle size dependent manner
Source: PLoS One. 2022 Sep 1;17(9):e0273852. doi: 10.1371/journal.pone.0273852 (PMC9436080; doi:10.1371/journal.pone.0273852)
Supplement: S1 File — (PDF) [file pone.0273852.s001.pdf]

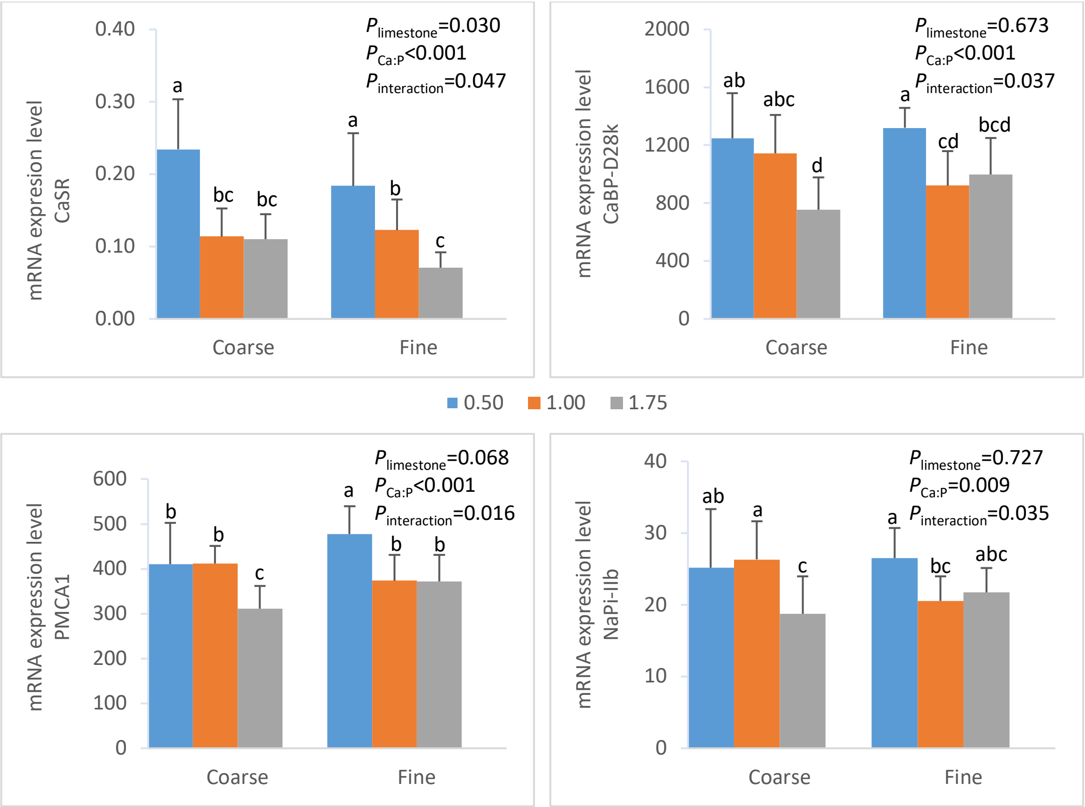

S1 Fig. Least square means of mRNA expression of calcium sensing receptor (CaSR), calbindin D28k (CaBP-D28k), plasma membrane Ca-ATPase 1 (PMCA1) and sodium-coupled phosphate transporter type IIb (NaPi-IIb) in the duodenal mucosa of broilers as affected by dietary total Ca : total P ratio (Ca:P), limestone particle size and their interaction.

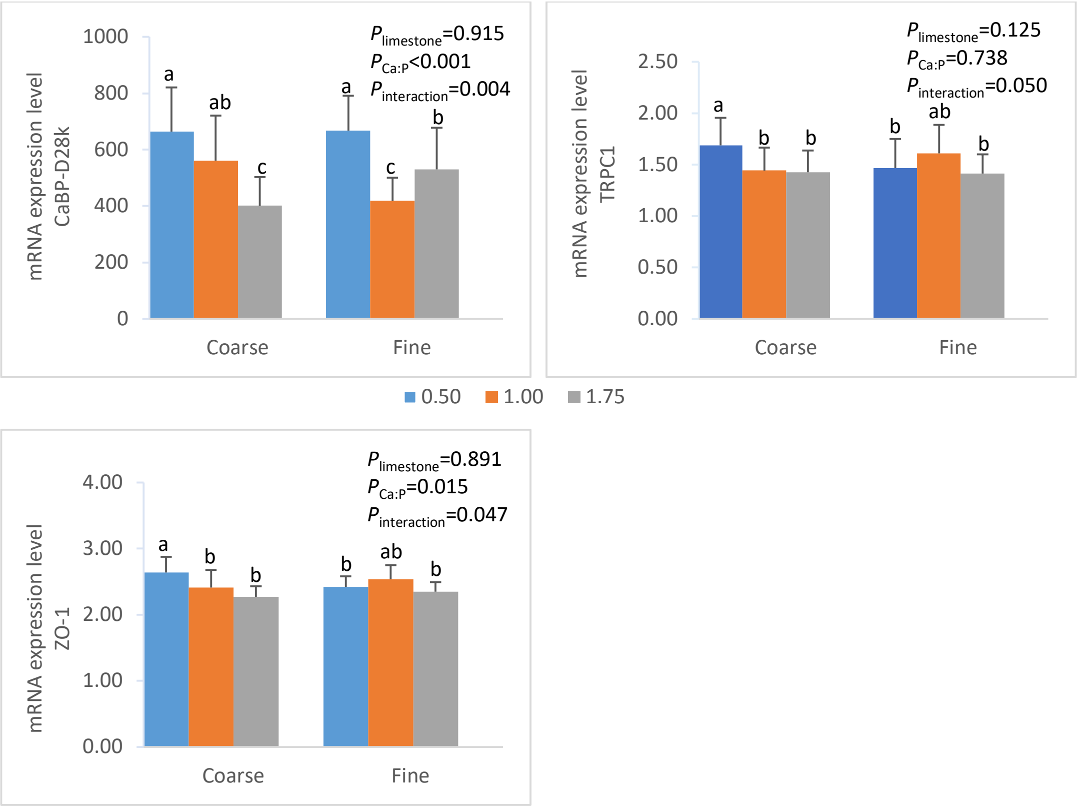

S2 Fig. Least square means of mRNA expression of calbindin D28k (CaBP-D28k), transient receptor potential canonical 1 (TRPC1) and zonula occludens-1 (ZO-1) in the jejunal mucosa of broilers as affected by dietary total Ca : total P ratio (Ca:P), limestone particle size and their interaction.
